# Integration of protein sequence and protein–protein interaction data by hypergraph learning to identify novel protein complexes

**DOI:** 10.1093/bib/bbae274

**Published:** 2024-06-08

**Authors:** Simin Xia, Dianke Li, Xinru Deng, Zhongyang Liu, Huaqing Zhu, Yuan Liu, Dong Li

**Affiliations:** School of Basic Medical Sciences, Anhui Medical University, 81 Meishan Road, Shushan District, Hefei 230032, China; State Key Laboratory of Medical Proteomics, Beijing Proteome Research Center, National Center for Protein Sciences (Beijing), Beijing Institute of Lifeomics, 38 Life Science Park, Changping District, Beijing 102206, China; State Key Laboratory of Medical Proteomics, Beijing Proteome Research Center, National Center for Protein Sciences (Beijing), Beijing Institute of Lifeomics, 38 Life Science Park, Changping District, Beijing 102206, China; State Key Laboratory of Farm Animal Biotech Breeding, College of Biological Sciences, China Agricultural University, 2 Yuanmingyuan West Road, Haidian District, Beijing 100193, China; State Key Laboratory of Medical Proteomics, Beijing Proteome Research Center, National Center for Protein Sciences (Beijing), Beijing Institute of Lifeomics, 38 Life Science Park, Changping District, Beijing 102206, China; State Key Laboratory of Medical Proteomics, Beijing Proteome Research Center, National Center for Protein Sciences (Beijing), Beijing Institute of Lifeomics, 38 Life Science Park, Changping District, Beijing 102206, China; School of Basic Medical Sciences, Anhui Medical University, 81 Meishan Road, Shushan District, Hefei 230032, China; State Key Laboratory of Medical Proteomics, Beijing Proteome Research Center, National Center for Protein Sciences (Beijing), Beijing Institute of Lifeomics, 38 Life Science Park, Changping District, Beijing 102206, China; School of Basic Medical Sciences, Anhui Medical University, 81 Meishan Road, Shushan District, Hefei 230032, China; State Key Laboratory of Medical Proteomics, Beijing Proteome Research Center, National Center for Protein Sciences (Beijing), Beijing Institute of Lifeomics, 38 Life Science Park, Changping District, Beijing 102206, China

**Keywords:** protein complex, hypergraph learning, protein sequence, protein network topology

## Abstract

Protein–protein interactions (PPIs) are the basis of many important biological processes, with protein complexes being the key forms implementing these interactions. Understanding protein complexes and their functions is critical for elucidating mechanisms of life processes, disease diagnosis and treatment and drug development. However, experimental methods for identifying protein complexes have many limitations. Therefore, it is necessary to use computational methods to predict protein complexes. Protein sequences can indicate the structure and biological functions of proteins, while also determining their binding abilities with other proteins, influencing the formation of protein complexes. Integrating these characteristics to predict protein complexes is very promising, but currently there is no effective framework that can utilize both protein sequence and PPI network topology for complex prediction. To address this challenge, we have developed HyperGraphComplex, a method based on hypergraph variational autoencoder that can capture expressive features from protein sequences without feature engineering, while also considering topological properties in PPI networks, to predict protein complexes. Experiment results demonstrated that HyperGraphComplex achieves satisfactory predictive performance when compared with state-of-art methods. Further bioinformatics analysis shows that the predicted protein complexes have similar attributes to known ones. Moreover, case studies corroborated the remarkable predictive capability of our model in identifying protein complexes, including 3 that were not only experimentally validated by recent studies but also exhibited high-confidence structural predictions from AlphaFold-Multimer. We believe that the HyperGraphComplex algorithm and our provided proteome-wide high-confidence protein complex prediction dataset will help elucidate how proteins regulate cellular processes in the form of complexes, and facilitate disease diagnosis and treatment and drug development. Source codes are available at https://github.com/LiDlab/HyperGraphComplex.

## Introduction

Protein–protein interactions (PPIs) form the foundation of numerous vital biological processes, with protein complexes being the key forms implementing these interactions [1]. Protein complexes, assembled from multiple protein subunits via non-covalent interactions [2], are large macromolecular complexes crucial for cellular homeostasis, intercellular interactions, signal transduction, as well as growth and proliferation [3–7]. For example, the 26S proteosome, comprising 31 distinct subunits, plays a crucial role in controlling the cell cycle, growth and apoptosis by degrading obsolete or damaged proteins [8]. Elucidating the components and functions of protein complexes is fundamental to comprehending cellular processes [9]. Additionally, diseases are often caused not by dysfunction of individual proteins but by dysregulated protein complex functions [10]. Researchers can infer relationships between drugs and diseases by studying protein complex compositions, facilitating new drug discovery [11]. In summary, the identification of protein complexes and their subunit composition is of great significance in elucidating complex biological processes within cells, advancing disease research and facilitating drug development.

To identify the composition of protein complexes via experimental methods is a labor-intensive and time-consuming task. The most common experimental technique for identifying protein complexes is tandem affinity purification coupled with mass spectrometry (TAP-MS) [12]. In brief, TAP-MS captures a target protein and all its binding partners by adding a TAP tag to the C-terminus of the bait protein and retrieving the complex with beads [13]. However, tagged proteins may interfere with complex formation and multi-step manipulations may cause transient low-affinity complexes to evade detection. In TAP-MS, in vitro purification of whole cell lysates loses subcellular localization information of complexes. Thus, time-consuming subcellular fractionation of lysates is required when studying specific cellular processes [14]. Given the limitations of experimental techniques for detecting protein complexes, developing an effective computational method for complex prediction is imperative.

In the past decade, the computational exploration of PPI networks to identify protein complexes has emerged as a primary approach in this field [15]. Existing protein complex identification methods can be roughly divided into three categories. (i) Methods based on network module detection: Numerous studies on PPI networks indicate the presence of modular structures [16], wherein certain regions of the network are more densely connected. These tightly connected modules often represent groups of proteins with shared functionality, i.e. potential protein complexes [17]. For instance, the Markov Clustering method divides the interaction network into disjoint dense clusters by simulating random walks in the network, thereby achieving module detection [18]. The ClusterOne method starts from individual seed nodes and employs a greedy growth process to incrementally add or remove nodes, identifying highly cohesive modules [19]. Such methods directly discover complex candidates in interaction networks based on the modular nature of protein complexes. (ii) Supervised methods based solely on network topology: These approaches leverage known protein complexes as training sets to extract features that distinguish real complexes from non-complexes. For example, Qi *et al.* extract network topology and biological features of known complexes, training a Bayesian model to score and filter candidate complexes [20]. Node2vec-RF learns protein embeddings in the network and uses them as input features to train a random forest (RF) model for classifying candidate complexes [21]. These methods, within a framework of feature engineering and supervised learning, can uncover more complex non-linear relationships between network and complex formation, addressing the limitations of network structure analysis. (iii) Methods based on multi-source information fusion: For instance, AdaPPI utilizes an adaptive graph convolution algorithm to compute protein functional similarities, subsequently constructing a weighted PPI network incorporating Gene Ontology (GO) attributes, thereby enhancing the accuracy of complex predictions [22]. These methods integrate multiple sources of information, such as network topology, functional annotation and expression data, overcoming the limitations of solely relying on network information for analysis and learning.

Despite the conceptual and performance breakthroughs achieved by the aforementioned algorithms in predicting protein complexes, several challenges persist. For instance: (i) Methods solely relying on PPI network for complex detection are highly influenced by network noise and cannot effectively predict small complexes or complexes with sparse internal connections; (ii) Current supervised learning methods often depend on feature engineering, requiring further research on adequately describing the biological features of protein complexes; (iii) Constrained by the review of domain experts, incomplete biological annotations for some proteins limit the effectiveness of multi-source information fusion methods.

In summary, current methods mainly focus on predicting protein complexes from a network topology perspective [15]. The amino acid sequence of a protein determines its three-dimensional structure, and different sequence order arrangements result in proteins possessing distinct physicochemical properties [23], which directly affects the biological functions of proteins and their binding abilities with other proteins [24]. Therefore, protein sequences play a key role in the formation of protein complexes. We believe that integrating protein sequence with PPI network topology to predict protein complexes is very promising, but effective algorithms for this integration are currently lacking.

We proposed a protein complex prediction model called HyperGraphComplex based on hypergraph learning. Hypergraph learning, which can represent higher-order non-pairwise complex relationships, rather than just one-to-one [25], and shows significant advantages over traditional graph convolutional neural networks, such as graph variational autoencoders (VGAE) [26], in learning more complex higher-order protein interaction patterns [27]. HyperGraphComplex integrates higher-order topology from the PPI network and protein sequence features by training an encoder and decoder simultaneously using a hypergraph variational autoencoder (HGVAE) to generate latent feature vectors of protein complexes, then combines them with a deep neural network (DNN) to identify candidate protein complexes. HyperGraphComplex does not rely on any manually crafted features and is entirely data-driven. To evaluate its performance, we conduct experiments on the yeast PPI network for comparison. Results show this method can effectively predict protein complexes in PPI networks, outperforming the state-of-the-art methods. Bioinformatics analysis demonstrated that the predicted complexes have similar biological attributes to known complexes. Remarkably, five of our predicted complexes, have recently been experimentally validated and reported in independent studies [28–32], with three of them exhibiting high-confidence structures using AlphaFold-Multimer (AFM).

## Material and methods

### Data preparation

#### Gold standard positive dataset

**Figure 1:**
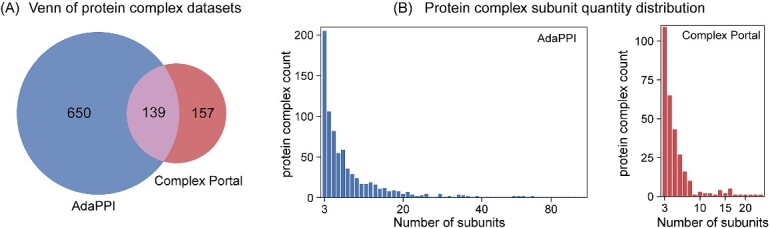
**The distribution of different complex datasets.** (A) Venn diagram illustrates the intersection of AdaPPI and complex portal complex datasets. (B) Distribution of the quantity of protein complex subunits in each of the two complex datasets: AdaPPI and complex portal. Note that all datasets adhere to a ‘power law’ distributions. Horizontal axis is the quantity of protein complex subunits. Vertical axis signifies the count of protein complexes that incorporate a specific number of subunits.

We utilized the known protein complexes containing three or more proteins included in the AdaPPI [22] as the positive training set, specifically including the MIPS [33], CYC2008 [34], Saccharomyces Genome Database (SGD) [35], Aloy [36] and TAP06 [37]. Additionally, we utilized yeast protein complexes from the Complex Portal database (downloaded in August 2023) [38] for independent test. These data sources include experimentally validated complexes obtained through manual literature curation (MIPS, CYC2008, SGD, Complex Portal), as well as complexes identified through experimental techniques such as TAP-MS (Aloy, TAP06). To objectively measure HyperGraphComplex’s performance, we excluded complexes from the Complex Portal database that overlapped with those in AdaPPI. This yielded 789 complexes for training and 157 for independent test (Figure 1A). The complex size distribution in AdaPPI and Complex Portal datasets followed a power-law distribution (Figure 1B). We further checked if these complexes come with 3D structures (hard-link), among the 946 protein complexes in this study, 56 complexes have annotated three-dimensional structural information available in the PDB database.

#### Gold standard negative dataset

We constructed the gold standard negative dataset based on the protocols of references Qi *et al*. [20] and Wang *et al*. [21]. Specifically, we randomly sampled nodes from the PPI network to form non-complexes, ensuring that the size distribution of these non-complexes follows the same power-law distribution as the known complexes in the gold standard positive dataset. Additionally, we maintained a ratio of 5:1 between the gold standard negative dataset and the gold standard positive dataset.

#### P‌PI interaction network

**Figure 2:**
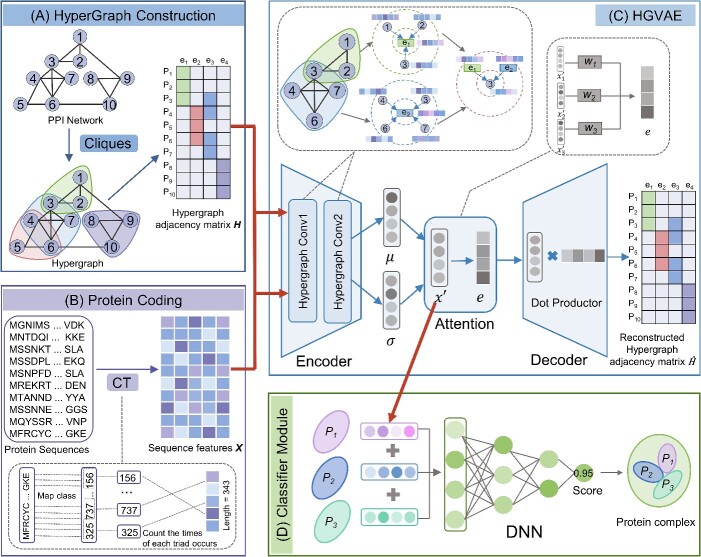
**The overall framework of HyperGraphComplex.** The model comprises of four components. (A) Hypergraph construction: This module takes the PPI network as input, uses cliques algorithm to extract protein fully connected subnet as hyperedge, and converts the PPI network into protein hypergraph. (B) Protein coding: This module takes the primary sequences of proteins as input and encode amino acid sequences of proteins using CT method and obtain protein sequence features $X$ (darker colors indicate higher conjoint triad frequency). (C) Self-supervised module for embedding protein sequence features: This module employs a hypergraph variational autoencoders (HGVAE) consisting of a hypergraph convolution encoder, an attention module and a dot product decoder, which is used to generate protein embeddings based on higher-order topological properties of hypergraph and the protein sequence features (see methods for details of σ, μ and *X’*). (D) Supervised module for predicting protein complex. The embeddings of given proteins are concatenated and utilized for predicting whether the given proteins can form a complex.

Statistical analysis of various yeast PPI datasets from different sources, encompassing protein nodes, interactions, cliques and protein complex coverage, was conducted (Table 1). To train our model with known complexes, we opted for PPI network datasets from databases with extensive complex coverage, specifically DIP [39], BIOGRID [40] and Mann-PPI [28]. Both the DIP database and BioGRID, which were downloaded from the AdaPPI resource, exclusively curate content from the literature, ensuring that they contain only high-reliability PPIs. Mann’s team constructed a highly reliable yeast global protein interaction network by employing affinity enrichment coupled to mass spectrometry, complemented by a quantitative two-dimensional analysis strategy [28]. Utilizing the Cliques percolation algorithm, we transformed the PPI network into a hypergraph with cliques as hyperedges (Figure 2A).

**Table 1:** Basic statistics of PPI datasets.

Dataset	Protein nodes	Interaction edges	Cliques	Protein complexes (subunits ≥ 3)
Collins	1622	9074	4763	311
Krogan-core	2705	7101	4399	325
Krogan14k	3544	13,959	9698	383
DIP	4928	17,201	13,523	679
BioGRID	5640	59,748	67,186	778
Mann	3927	31,004	17,184	558

#### Protein sequence encoding

Protein sequences sourced from UniProt [41] were encoded using the conjoint triad (CT) method [42], a widely employed approach for amino acid sequence encoding. The CT method categorizes the 20 amino acids into 7 classes based on dipoles and side chain volumes, as outlined in Supplementary Table 1, to mitigate dimensionality and account for synonymous mutations. Next, a sliding window of size 3 is moved through each protein sequence to compute the frequency of each triad type, capturing properties of individual amino acids and their adjacent amino acids (Figure 2B). CT encoding obtains fixed dimensional representations of amino acid sequences with 343 (7 × 7 × 7) dimensions.

### Hypergraph variational autoencoder

Inspired by graph domain representation learning tasks, we proposed a hypergraph variational autoencoder (HGVAE) model for hypergraph representation learning. The model design involves two steps, which can be understood as a decoding operation, as shown in Figure 2C. The method simultaneously trains an encoder and decoder. The encoder integrates node information and its neighborhood via hyperedge-node association, using hypergraph Laplacian smoothing filters [43]. Latent representations X’ are then formed by introducing a standard Gaussian distribution through variational inference. The decoder focuses on the relationships between node and hyperedge features while preserving the hypergraph structure to learn expressive low-dimensional node embeddings.

First, in this study, we treated the PPI network as an undirected attributed hypergraph $G=\left(V,E,X,W\right)$, where V denotes the node set, E denotes the hyperedge set, X denotes the feature matrix and W denotes the diagonal matrix composed of hyperedge weights. The feature matrix $X\in{R}^{n\ast d}$ consists of the feature vectors of all nodes, where n is the number of nodes and d is the dimension of node features. v_i_ is used to denote a protein node in the PPI network, and e_k_ represents a fully connected subgraph in the PPI network. For the hypergraph G, an incidence matrix $H=\mid V\mid \ast \mid E\mid$ is used to describe the relationships between nodes and hyperedges, where |E| is the number of hyperedges. Each element $h\left(i,k\right)$ in H, indicating whether node v_i_ belongs to hyperedge e_k_, is calculated as:


(1) \begin{equation*} h\left(\mathrm{i},k\right)=\left\{\begin{array}{c}1, if\ {v}_i\in{e}_k\\{}0, if\ {v}_i\notin{e}_k\end{array}\right. \end{equation*}

We defined a hypergraph convolution function ${f}_{hgnn}$ [43]:


(2) \begin{equation*} {X}^{\left(l+1\right)}={f}_{hgnn}\left({X}^{(l)},H,{\varTheta}^{(l)}\right) \end{equation*}

Here, we utilized the input ${X}^{(l)}$ in a hypergraph convolutional operation, yielding the output ${X}^{\left(l+1\right)}$. The hypergraph matrix H serves as the kernel for this calculation. In our work, protein sequence features matrix X serves as the initial matrix. The individual layers of our hypergraph convolutional network [43] can be defined as follows:


(3) \begin{equation*} {f}_{hgnn}\left({X}^{(l)},H,{\varTheta}^{(l)}\right)= ReLU\left({D}_v^{-1/2} HW{D}_e^{-1}{H}^T{D}_v^{-1/2}{X}^{(l)}{\varTheta}^{(l)}\right) \end{equation*}

where ${\varTheta}^{(l)}\in{\mathbb{R}}^{d_l\times{d}_{l+1}}$. ${d}_l$ is the dimension of input for convolution, ${d}_{l+1}$ is the dimension of output after convolution. D_v_ is the degree matrix associated with edges. D_e_ is the degree matrix associated with vertices. Our hypergraph encoder consists of two HGNN layers, and we let the prior over the latent variables *X’* be the centered isotropic multivariate Gaussian [44]:


(4) \begin{equation*} q\left({X}^{\prime }|X,H\right)={\prod}_{i=1}^nq\left({x}_i^{\prime }|X,H\right) \end{equation*}

(5) \begin{equation*} q\left({x}_i^{\prime }|X,H\right)=\mathcal{N}\left({x}_i^{\prime }|{\mu}_i,{\operatorname{diag}}\left({\sigma}_i^2\right)\right) \end{equation*}

In our study, we defined the prior over the latent variables ${X}^{\prime }$ as a centered isotropic multivariate Gaussian distribution with mean $\mu$ and standard deviation $\sigma$.


(6) \begin{equation*} {z}_i={\mu}_i+{\sigma}_i\bigodot{\epsilon}_i \end{equation*}

where $\bigodot$ is element-wise multiplication and ${\epsilon}_i\sim \mathcal{N}\left(0,1\right)$.

We calculated the attention weights for each hyperedge in the hypergraph. For a hyperedge, the formula for computing its attention weights is: 


(7) \begin{equation*} {w}_i= Softmax\Big( projection\left({X}_i^{\prime}\right) \end{equation*}

Where ${X}_i$ is the set of node features corresponding to hyperedge e, and the projection function, consist of two linear layer modules:


(8) \begin{equation*} projection\left({X}_i^{\prime}\right)=L\mathrm{e} akyReLU\left({W}_2\left( leakyReLU\left({W}_1{X}_i^{\prime }+{b}_1\right)\right)+{b}_2\right) \end{equation*}

The hyperedge feature vector ${e}_i$:


(9) \begin{equation*} {e}_i=L\mathrm{e} akyReLU\left({\sum}_{j=1}^{N_i}{w}_{ij}\bullet{X}_{ij}^{\prime}\right) \end{equation*}

Next, we described a basic inner product decoder that aims to reconstruct *H* using the learned latent variable *X’*:


(10) \begin{equation*} p\left(\hat{H}|{X}^{\prime },E\right)={\prod}_{i=1}^n{\prod}_{j=1}^mp\left({\hat{H}}_{ij}|{x}_i^{\prime },{e}_j\right) \end{equation*}

(11) \begin{equation*} p\left({\hat{H}}_{ij}=1|{x}_i^{\prime }{e}_j\right)= sigmoid\left({x}_i^{\prime },{e_j}^T\right) \end{equation*}

Finally, to maximize the similarity between the reconstructed hypergraph matrix $\hat{H}$ and the hypergraph matrix $H$, we optimized the model by minimizing the following loss function:


(12) \begin{equation*} \mathcal{L}={\mathbb{E}}_{q\left({X}^{\prime }|H\right)}\left[{\log}p\left(\hat{H}|{X}^{\prime },E\right)\right]- KL\left[q\left({X}^{\prime }|H\right)\parallel p\left({X}^{\prime}\right)\right] \end{equation*}

In this study, the Kullback–Leibler divergence, $KL\left[q\left(\cdotp \right)\parallel p\left(\cdotp \right)\right]$, is employed to quantify the dissimilarity between the distributions $q\left(\cdotp \right)$ and $p\left(\cdotp \right)$ [45]. As $p\left({X}^{\prime}\right)$ is assumed to follow a normal distribution with mean 0 and standard deviation 1 (i.e. $p\left({X}^{\prime}\right)\sim \mathcal{N}\left(0,1\right)$), the cost function represents the capability of the model in reconstructing the input network and aligning the latent variables with $p\left({X}^{\prime}\right)$. The optimization of the cost function with respect to the parameters of the encoder is performed using stochastic gradient descent.

### Classifier module

A protein complex is a set of proteins. We calculate the vector of protein complex according to the vector representation of the protein. The calculation method is shown in formula [21].


(13) \begin{equation*} complex\left({\varphi}_1,{\varphi}_2,\dots, {\varphi}_m\right)= avgZ\left(.,j\right)\ 0\le j<d \end{equation*}

where ${\varphi}_i\ \left(\mathrm{i}=1,2,\dots, \mathrm{m}\right)$ is the vector representation of protein nodes in a protein complex. *Z* is the matrix composed of the vector representation ${\varphi}_i$of protein nodes in a protein complex, d is the dimension of ${\varphi}_i$ and $Z\left(.,j\right)$- is the j-th column in matrix Z.

**Figure 3:**
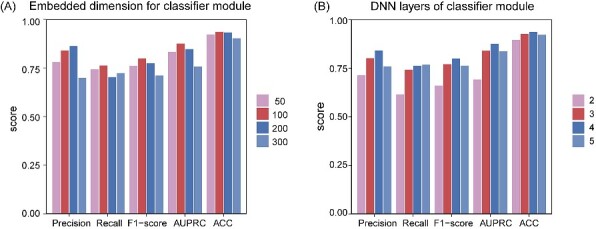
**The influence of embedding dimension and layer quantity on the performance of the HyperGraphComplex classifier module.** (A) Demonstrates the effect of varying the embedding dimension, while (B) elucidates the impact of altering the number of layers within the HyperGraphComplex classifier module.

DNN is widely recognized as a powerful and prevalent method for supervised learning [46]. We input the feature vectors of protein complexes into a DNN for predicting the likelihood of diverse subunit compositions within protein complexes. Figure 3 shows the effect of different embedding dimensions and DNN layers on the experimental results. In this study, the dimension of the embedding vector of the protein complex is 100. DNN consists of four layers, with batch normalization and dropout implemented between each layer.

### Model evaluation

We evaluate the performance of HyperGraphComplex and compare with state-of-the-art complex prediction models via the following metrics (Precision, Recall, F1-score based on neighborhood affinity score (NA) and complex-wise accuracy (Acc)) that are commonly used in the complex prediction [47, 48]. NA(p, b) is calculated between a predicted complex p and a known complex b as follows:


(14) \begin{equation*} NA\left(p,b\right)=\frac{{\left|{\mathcal{V}}_p\cap{\mathcal{V}}_b\right|}^2}{\mid{\mathcal{V}}_p\mid \times \mid{\mathcal{V}}_b\mid } \end{equation*}

Precision is calculated as the proportion of the number of matched predicted complexes (N_cp_) to the total number of predicted complexes (P):


(15) \begin{equation*} \left\{\begin{array}{@{}c}{N}_{cp}=\mid \left\{p|p\in P,\exists b\in B, NA\left(p,b\right)\ge 0.25\right\}\mid \\{} Precision=\frac{N_{cp}}{\mid P\mid}\end{array}\right. \end{equation*}

Recall is the proportion of the number of matched known complexes (N_cb_) to the number of known ones (B):


(16) \begin{equation*} \left\{\begin{array}{@{}c}{N}_{cb}=\mid \left\{b|b\in B,\exists p\in P, NA\left(p,b\right)\ge 0.25\right\}\mid \\{} Recall=\frac{N_{cb}}{\mid B\mid}\end{array}\right. \end{equation*}

F1-score is accordingly determined by


(17) \begin{equation*} F1- score=\frac{2\times Precision\times Recall}{Precision+ Recall} \end{equation*}

The three aforementioned metrics are computed based on the number of matched modules [22]. The complex-wise accuracy (Acc), is utilized to highlight the number of matched proteins. Different from the other three metrics, Acc does not set a threshold for a module using the matched protein proportion. Acc is calculated as follows:


(18) \begin{equation*} Acc=\sqrt{Sn\times PPV} \end{equation*}

where the complex-wise sensitivity (Sn) is determined by


(19) \begin{equation*} Sn=\frac{\sum_{i=1}^{\mid B\mid}\kern0.1em {{\max}}_{j=1}^{\mid P\mid}\kern0.1em \left\{{T}_{ij}\right\}}{\sum_{i=1}^{\mid B\mid}\kern0.1em {N}_i} \end{equation*}

and the complex-wise predictive positive value is formulated as


(20) \begin{equation*} PPV=\frac{\sum_{j=1}^{\mid P\mid}\kern0.1em ma{x}_{i=1}^{\mid B\mid}\left\{{T}_{ij}\right\}}{\sum_{j=1}^{\mid P\mid}\kern0.1em \sum_{i=1}^{\mid B\mid}\kern0.1em {T}_{ij}} \end{equation*}

T_ij_ represents the intersection of proteins between known complex i and predicted complex j. N_i_ denotes the number of proteins in the known complex i. Additionally, we evaluate classifier module performance using ROC and PR curves, calculating AUROC and AUPRC values. We performed 30 random sampling evaluations on the negative data set and calculated the means and standard errors of evaluation metrics.

### Identification of protein complex by HyperGraphComplex

To identify as many complexes as possible, we devised a mining algorithm leveraging the PPI network from Mann *et al.* The process involves two steps: (i) Expanding each PPI in the network by HyperGraphComplex. We find the set of all neighbor nodes for each PPI, add them individually to the PPI and use the model to score it to find the highest scoring neighbor node above a model threshold α to form a new subgraph. Then we add the remaining neighbors to the new subgraph, repeat the above process until no introducible nodes lead to a score higher than the threshold. (ii) Merging candidate complexes. Candidate complexes are sorted in descending order by their scores by HyperGraphComplex. For each candidate, we calculate its overlap with all lower scoring candidates. If the overlap is higher than threshold β, and their combined score is higher than the individual score, a merge operation would be performed, otherwise we remove the lower scoring candidate. The algorithm is outlined in Algorithm 1.

**Algorithm 1:** Protein complex detection algorithm.

**Input:** PPI network *G*, model *M*, thresholds *α, β*
**Output:** Predicted protein complex set *P*
1: //Find the candidate complex set *C_S_*
2: **for** each *PPI* in *G* **do**
3: Collecting the set of neighbors for *PPI*, denoted as *N(PPI)*
4: **if** *N(PPI)* > 0 **then**
5: MaxScore *S* ← 0
6: *Condition* ← *TRUE*
7: **while** *Condition* **do**
8: ** for** *v_i_* **in** *N(PPI)* **do**
9: new subgraph *NSg* ← *Null*
10: subgraph *Sg* ← *PPI* ∪ *v_i_*
11: subgraph score *M_S_* ← **model** *M(Sg)*
12: **if** *M_S_* > *α* **and** *M_S_* > *S* **then**
13: MaxScore *S ← M_S_*
14: new subgraph *NSg ← Sg*
15: **end if**
16: **end for**
17: *N*(*PPI*) *← N*(*PPI*) - *NSg*
18: **if** *NSg* is *Null* **then**
19: *Condition ← FALSE*
20: **else**
21: *PPI ← NSg*
22: ** end if**
23: ** end while**
24: ** end if**
25: Add *PPI* to candidate complex set *C_S_*
26: **end for**
27: //Merge the candidate complexes
28: candidate complex scores *M_S_ ←* **model** *M*(*C_S_*)
29: rank the candidate complex set *C_S_* in descending order of *M_S_*
30: **for** *c_i_* in *C_S_* **do**
31: ** for** *c_j_* **in** *C_S_* **where** *j > i* **do**
32: ** if overlap**(*c_i_*, *c_j_*) *> β* **and** *M*(*c_i_* ∪ *c_j_*) *> M*(*c_i_*) **then**
33: *c_i_* = *c_i_* ∪ *c_j_*
34: ** end if**
35: **end for**
36: **if** nodes number *N(c_i_) > 2 **then***
37: Add *c_i_* to predict complex set *P*
38: ** end if**
39: **end for**
40: **return** *P*

### GO semantic similarity analysis

We performed GO semantic similarity analysis and differential analysis on known complexes, predicted complexes and randomly sampled protein sets to validate the reliability of the predicted complexes. We first collected the GO terms for each protein in a predicted complex based on annotations in the UniProt database (i.e. GO resource) [41]. Then, for every possible protein pair, we calculated pairwise GO term semantic similarity scores using the R package GOSemSim [49, 50]. For example, given a protein complex with three subunits a, b and c, all the GO terms are collected, including Molecular Function (MF), Biological Process (BP) and Cellular Component (CC). For each GO category and the three possible protein pairs including a-b, a-c and b-c, the semantic similarity scores of all pairwise GO terms are computed and the average score is reported as the total score for the current GO category. In this case, the various GO scores for the protein complex are defined as [51]:


(21) \begin{equation*} GO: BP=\frac{BP\left(a,b\right)+ BP\left(b,c\right)+ BP\left(a,c\right)}{3} \end{equation*}

(22) \begin{equation*} GO: CC=\frac{CC\left(a,b\right)+ CC\left(b,c\right)+ CC\left(a,c\right)}{3} \end{equation*}

(23) \begin{equation*} GO: MF=\frac{MF\left(a,b\right)+ MF\left(b,c\right)+ MF\left(a,c\right)}{3} \end{equation*}

### Expression concordance analysis

We assessed the expression coherence of complex members by calculating pairwise Manhattan distances between proteins using the mass spectrometry data provided in Karayel *et al.*’s study [52]. Firstly, we computed the Manhattan distance between each interacting protein pair using the following formula [53]:


(24) \begin{equation*} {D}_{ij}=\left|{M}_{i.}-{M}_{j.}\right| \end{equation*}

Where D_i,j_ denotes the Manhattan distance between protein pair i. and j. M_i._ and M_j._ are the rows of matrix M corresponding to the abundances of protein i and protein j across all samples, respectively. Subsequently, based on the Manhattan distance matrix of protein pairs, we assessed the distances between subunits within protein complexes.

### GO enrichment analysis

We performed GO enrichment analysis using the R package ClusterProfiler [54] to determine if members of the predicted complexes share common GO terms. The p-value indicates the statistical significance of the predicted protein functional modules relative to given functional annotations, calculated using the following hypergeometric distribution [22]:


(25) \begin{equation*} P- value=1-\sum_{i=0}^{t-1}\frac{\left(\begin{array}{c}\left|F\right|\\{}i\end{array}\right)\left(\frac{\left|V\right|-\left|F\right|}{\left|C\right|-\mathrm{i}}\right)}{\left(\begin{array}{c}\left|V\right|\\{}\left|C\right|\end{array}\right)} \end{equation*}

Where |C| denotes the number of proteins identified in a complex C by the algorithm, i represents the number of proteins containing a function F in the identified complex C, |F| indicates the number of proteins in the protein network containing that function and |V| denotes the number of protein nodes contained in the PPI network. For multiple different GO terms, some complexes may have lower p-values. We selected the best (lowest *P*-value) GO term for each complex.

## Results and discussion

### Overview of HyperGraphComplex

The overall framework of the proposed HyperGraphComplex is shown in Figure 2, which consists of four main modules: hypergraph adjacency matrix construction, protein sequence feature encoding, extracting protein features via HGVAE and classifier module of protein complexes. First, the ordinary graph structure of the PPI network is converted into a hypergraph structure by utilizing maximum cliques searched in the PPI network by the Cliques algorithm [55] as hyperedges, to construct the hypergraph adjacency matrix. Protein sequences are encoded into fixed-dimensional initial feature vectors using the CT method [42]. Meanwhile, HyperGraphComplex performs hypergraph structure learning on the PPI network via the designed HGVAE, which integrates higher-order topology from the PPI network and protein sequence features by training an encoder and decoder simultaneously to generate latent feature vectors for proteins. The encoder module finds global semantic neighbors via two hypergraph convolution layers to supplement local structure information, while simultaneously learning the parameters of a probabilistic distribution for the latent representations. Node latent representations are then sampled from the learned distribution using variational inference. In the decoder, latent representations of nodes in hyperedges are synthesized into hyperedge latent representations using a self-attention mechanism. The decoder takes node and hyperedge latent representations to reconstruct the hypergraph adjacency matrix. Finally, the classifier module concatenates the above learned protein latent feature vectors to form an embedding vector for complexes, and feeds them into a fully connected DNN. Leveraging the well-designed modules above, HyperGraphComplex predicts potential protein complexes in PPI networks with the state-of-the-art performance.

### Performance comparison with other methods

To demonstrate the reliability of the HyperGraphComplex model, we compared it with several mainstream methods. Among them, ClusterOne [19] and AdaPPI [22] are methods that find complexes by dense subgraph mining in unweighted or weighted PPI networks. Node2vec-RF obtains feature vectors of proteins via network representation learning based on random walks, then trains a RF model to identify candidate complexes. For comparison with Node2vec-RF, we chose the best embedding dimension and parameters reported in the study [21], and retrained Node2vec-RF on our training dataset. To enable a fair comparison, we leveraged the identical PPI network datasets, protein complex benchmarks and same evaluation metrics utilized in their studies.

As shown in Table 2, HyperGraphComplex is significantly superior to the other methods on four metrics across the two PPI databases. Compared to Node2vec-RF based on network topological features, the Acc on the DIP database is improved by 17.31%; on BioGRID database Acc improved by 17.48%. In summary, HyperGraphComplex outperforms the other comparative methods on evaluation metrics including F1-score and Acc, fully demonstrating that HyperGraphComplex can more accurately and with higher coverage capture protein complexes in PPI networks by integrating higher-order topological features of PPI networks and sequence features of proteins. Additionally, to further test the model’s predictive capability, we combined all soft-link complexes (without known 3D structures) to construct a new training set comprising 890 complexes for model retraining, and evaluated our model separately on the 56-complex hard-link test set (with 3D structures). HyperGraphComplex performs well on complexes with known structures (Supplementary Table 2), even when no hard-link complexes were included in the training set.

**Table 2:** Performance on protein complex identification in two PPI datasets.

Method	DIP				BioGRID			
	F1-score	Precision	Recall	Acc	F1-score	Precision	Recall	Acc
ClusterOne	0.365	0.390	0.343	0.227	0.439	0.393	0.497	0.316
AdaPPI	0.583	0.675	0.514	0.259	0.566	0.600	0.535	0.303
Node2vec-RF	0.426	0.312	0.675	0.312	0.452	0.326	0.735	0.326
**HyperGraphComplex**	**0.761**	**0.740**	**0.785**	**0.366**	**0.822**	**0.816**	**0.830**	**0.383**

We further expanded HyperGraphComplex to the prediction of human protein complexes (Supplementary Table 3). HyperGraphComplex outperforms the baseline PC2P [56] method in terms of F-score and Acc. These results demonstrate that HyperGraphComplex exhibits good applicability and generalization capability across different organisms.

### Ablation experiments

**Figure 4:**
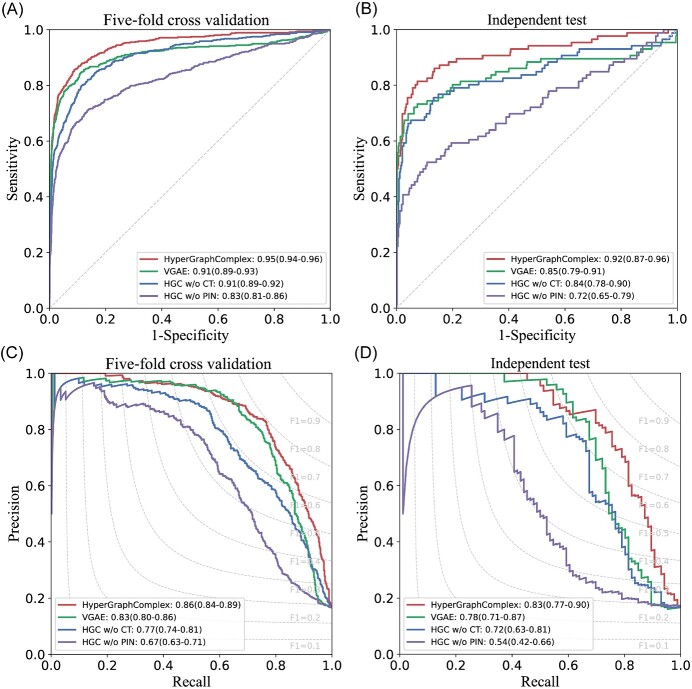
**Performance evaluation and comparison of HyperGraphComplex with three other HyperGraphComplex’s variants.** To independently evaluate the impact of both features and model framework used by HyperGraphComplex, we constructed three model variants: (i) VGAE: Substituting HGVAE with VGAE to integrate sequence features and PPI network topology for predicting protein complexes; (ii) HGC w/o PIN: Removing PPI network topology from HyperGraphComplex, and only sequence features were used; (iii) HGC w/o CT: Removing sequence features encoded by CT method from HyperGraphComplex, and only PPI network topology was used. Employing 5-fold cross-validation and independent test, we found that HyperGraphComplex, which encompasses both sequence features and the hypergraph framework, demonstrated the highest prediction performance among the four models. The ROC curves of the assessment models demonstrate sensitivity and specificity (A, B) and the PR curves of the assessment models precision and recall (C, D) against a particular prediction score cutoff, with each point on the curves representing the respective values. The labels on each panel correspond to the area under the curve along with its 95% confidence interval. The reference lines indicate non-informative predictions with an AUROC of 0.5 (A, B) or predictions with a constant F1 score across different thresholds (C, D).

To ascertain the decisive impact of sequence features and the hypergraph framework on the predictive performance of HyperGraphComplex, we constructed three variants of HyperGraphComplex and conducted ablation experiments on the Mann-PPI dataset: VGAE, which we substituted HGVAE with VGAE to integrate sequence features and PPI network topology for predicting protein complexes; HyperGraphComplex without PPI network topology (HGC w/o PIN), which we removed PPI network topology from HyperGraphComplex, and only sequence features were used; HyperGraphComplex without CT (HGC w/o CT), which we removed sequence features encoded by CT method from HyperGraphComplex, and only PPI network topology was used. The comparison results indicate that both sequence features and the hypergraph framework are paramount for enhancing predictive efficacy (Table 3, Figure 4).

**Table 3:** Performance of HyperGraphComplex with three model variants in Mann-PPI dataset^*^.

Method	F1-score	Precision	Recall	Acc
**HyperGraphComplex**	**0.815 (±0.002)**	0.804 (±0.006)	**0.827 (±0.004)**	**0.427 (±0.002)**
VGAE	0.801 (±0.003)	**0.821 (±0.007)**	0.784 (±0.004)	0.426 (±0.002)
HGC w/o CT	0.758 (±0.003)	0.755 (±0.007)	0.762 (±0.004)	0.406 (±0.002)
HGC w/o PIN	0.723 (±0.004)	0.726 (±0.009)	0.723 (±0.004)	0.420 (±0.001)

*Note*: VGAE: Substituting HGVAE with VGAE to integrate sequence features and PPI network topology for predicting protein complexes; HGC w/o PIN: Removing PPI network topology from HyperGraphComplex, and only sequence features were used; HGC w/o CT: Removing sequence features encoded by CT method from HyperGraphComplex, and only PPI network topology was used.

Specifically, when protein sequence features were disregarded, and predictions were solely based on the PPI network topology obtained from HGVAE, there was a significant decrease in various evaluation metrics (Table 3, Figure 4). This strongly demonstrates the indispensability of sequence features for accurately predicting the composition of protein complexes.

In addition, to validate the necessity of using the hypergraph framework for predicting complex composition, we replaced the hypergraph framework in HyperGraphComplex with a conventional graph learning method (VGAE) [26]. We observed that the performance of VGAE was significantly lower than that of HyperGraphComplex, indicating that the hypergraph framework indeed better integrates the topological properties of PPIs and protein sequence information. In summary, the comprehensive sequence features, the PPI network topology and hypergraph framework collectively determine the outstanding performance of HyperGraphComplex in predicting protein complex composition.

To evaluate the robustness of HyperGraphComplex to false positive (randomly sampled non-interacting protein pairs) and false negative (randomly removed true PPIs) within the training data, we separately introduced these two types of noise samples into the Mann-PPI dataset, and retrained and reevaluated our model. We found that HyperGraphComplex exhibited good robustness, with limited performance degradation across various evaluation metrics (Supplementary Tables 4 and 5). This demonstrates the algorithm’s ability to make efficient predictions even with noisy training data.

### Protein complex biological property analysis

**Figure 5:**
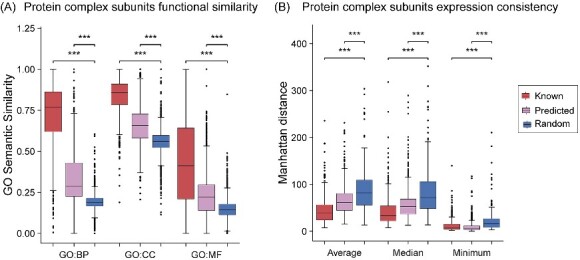
**Multidimensional association features for known protein complexes, predicted protein complexes, and randomly generated pseudo protein complexes.** The feature scores include GO term similarity in terms of biological process (BP), cellular component (CC) and molecular function (MF) (A) and the average, median, and minimum Manhattan distance of the protein complexes, calculated based on the abundance of their subunits (B). Wilcoxon test is used to test the difference between random pseudo protein complexes and known or predicted protein complexes. Our results show that for all kinds of features, the average score of random pseudo protein complexes is significantly lower than that of known and predicted protein complexes (^*^^*^^*^  *P*-value <0.001).

Proteins within complexes often exhibitspatiotemporal co-expression and co-localization, as proteins with shared or analogous functions tend to form complexes through synergistic interactions to jointly regulate physiological processes or reactions [53, 57]. This synergy stems from subunits’ expression patterns being influenced by similar signals and regulatory mechanisms. Co-expression enhances complexes’ efficiency and stability in cellular functions and signal transduction [58]. To evaluate the quality of the predicted complex dataset, we performed GO semantic similarity assessments and expression consistency analyses. Specifically, we randomly generated pseudo protein complexes based on the predicted complex subunit distribution, and calculated the GO semantic similarity and Manhattan distance among the random pseudo protein complexes, predicted complexes, and known complexes (Figure 5). The results demonstrated that real complexes and predicted complexes differ significantly from random pseudo protein complexes in GO semantic similarity and Manhattan distance. This implies predicted complexes are not random protein assemblies but rather possess functional synergy and expression consistency, consistent with the characteristics of real complexes. Additionally, GO enrichment analysis of predicted complexes indicates that the subunits of the predicted complexes can be enriched in the same GO terms. Therefore, we can conclude our predicted complexes are biologically meaningful and warrant further investigation.

### Case study: Complexes recognized by HyperGraphComplex influence key biological processes

To verify whether HyperGraphComplex can accurately predict potential protein complexes, we selected several complexes with certain biological significance and utilized HyperGraphComplex to predict additional proteins which can form stable novel complexes with these known ones. These novel complexes are crucial for a comprehensive understanding of cellular regulatory networks.

Protein kinase CK2 is a highly conserved serine/threonine protein kinase ubiquitously presents in eukaryote [59]. Yeast CK2 consists of two catalytic subunits (Cka1, Cka2) and two regulatory subunits (Ckb1, Ckb2), and disruption of both catalytic subunits leads to loss of enzymatic activity [60]. CK2 can phosphorylate various protein substrates, such as Atg32 [61], Tom22, Mim1 [62], Swi6 [63] and Sir2 [64], thereby participates in regulating various physiological processes including cell proliferation, differentiation and apoptosis [65]. Different proteins can stably interact with CK2 to form complexes, altering kinase structure and activity, enabling its involvement in various cellular processes in response to diverse cellular contexts [66]. Dysregulated CK2 activity has been observed in various diseases, especially cancers, leading to lack of phosphorylation of some key substrates [67]. Thus, CK2 has become an important drug target. Some CK2 inhibitors are currently in clinical trials, but off-target effects exist [68].

**Figure 6:**
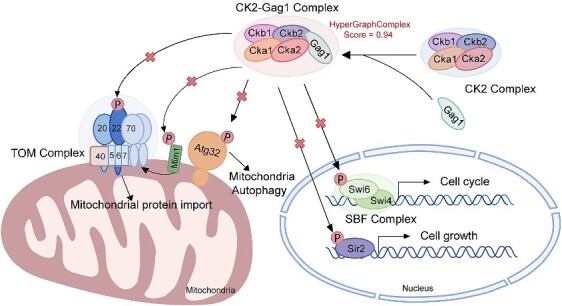
**The CK2-Gag1 complex recognized by HyperGraphComplex affects various biological processes.** Protein kinase CK2 phosphorylates various substrates to regulate cellular processes. Specifically, CK2 facilitates mitochondrial autophagy by phosphorylating Atg32. Additionally, CK2 promotes biogenesis of the TOM complex by phosphorylating Tom22 and Mim1, thereby influencing mitochondrial protein import. CK2 directly phosphorylates Swi6, participating in regulating G1/S gene transcription. CK2 also affects cell growth by regulating phosphorylation of the NAD-dependent protein deacetylase Sir2. Finally, we hypothesize formation of the CK2-Gag1 complex may alter CK2 structure, impacting its phosphorylation activity, affecting yeast cellular physiological processes. Scores in the figure are prediction confidence scores assigned by HyperGraphComplex.

To further investigate CK2-related complexes in cells, we predicted several potential proteins which can form stable complexes with CK2 using HyperGraphComplex. Gag1, predicted with a relatively high score of 0.94, was validated by the experiment in Mann’s study [28]. Mann *et al.* found that overexpression of Gag1 in yeast strains caused growth defects. This is likely because the CK2-Gag1 complex alters the original kinase structure of CK2, preventing phosphorylation of some substrate molecules (Figure 6). This suggests the CK2-Gag1 complex may impact key CK2-mediated biological processes, providing new insights into regulatory networks in cells. Moreover, since CK2 is highly conserved, potential human homologs of yeast Gag1 may also form complexes with human CK2. Therefore, studying the yeast CK2-Gag1 mechanism contributes to the study of CK2-related diseases and provides insights for the development of safer and more effective CK2-targeted drugs.

Moreover, Mco6 (score:0.98) was predicted to form a novel complex with the SAM^Mdm10^ complex. Busto *et al.* validated this complex (SAM^Mco6^ complex) and found it is crucial for the efficient assembly of the main mitochondrial protein entry gate TOM complex [29]. Sem1 (score:0.82) was predicted to form a novel complex (Thp3-Csn12-Sem1 complex) with the Thp3-Csn12 complex, which was experimentally verified by independent study [30]. During transcription, the Thp3-Csn12-Sem1 complex first captures the pre-mRNA, directing it to the spliceosome for mRNA splicing, which was also experimentally verified [30]. HyperGraphComplex identified Atg18 (score:0.93) to form a novel complex with the CSC complex. Courtellemont *et al.* validated this prediction and reported that the novel CROP complex plays a critical role in cellular component trafficking [31, 69]. Kar4-Vir1-Dyn2 (score:0.82) was predicted as novel subunits of the MTC complex, which was validated by independent study and was found to be involved in regulating m6A modification of mRNA [32, 70].

**Figure 7:**
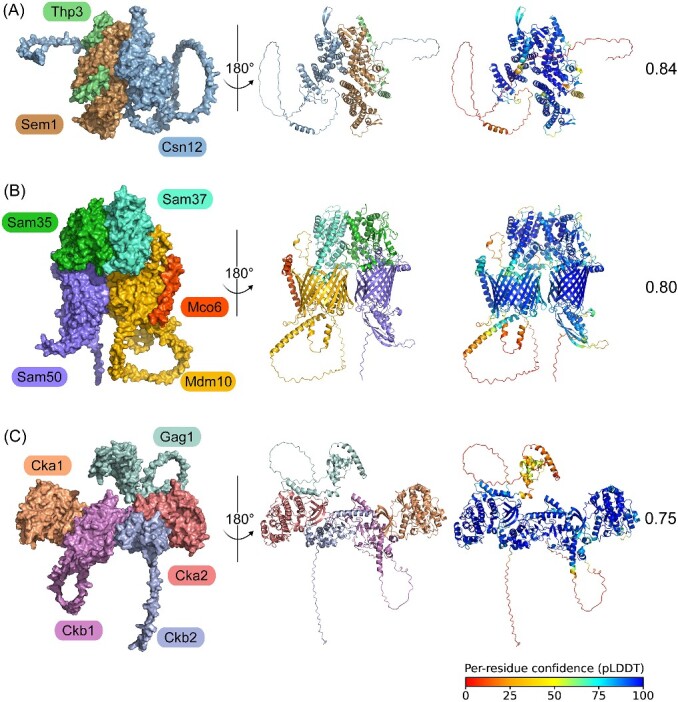
**Selection of high-confidence complex structures.** Best structures predicted by AlphaFold-Multimer (AFM) for these complexes were validated by independent study, with their ipTM + pTM scores ranging from 0 (worst) to 1 (best). (A) AFM-predicted model of Thp3, Sem11, and Csn12. Left: Surface representation with distinct subunits color-coded. Middle: Structure rotated 180 degrees around the central axis, depicted as cartoons. Right: Complex colored using a rainbow scheme based on pLDDT scores, ranging from 0 (worst) to 100 (best). (B) AFM-predicted model of Sam37, Sam35, Sam50, Mdm10 and Mco6, presented similarly to (A). (C) AFM-predicted model of Cka1, Ckb1, Ckb2, Cka2, and Gag1, presented similarly to (A). pTM, predicted template modeling score; ipTM, interface pTM.

We further utilize AFM version 2.3.0 to generate the structures of these complexes and assess their stability using the ipTM + pTM score [28, 71, 72]. We found the structures of three protein complexes exhibit high confidence (CK2-Gag1 complex, SAM^Mco6^ complex, Thp3-Csn12-Sem1 complex). PyMOL was used to showcase the predicted protein complex structures (Figure 7). These results increase the reliability of predicted results of HyperGraphComplex framework.

## Conclusion and discussion

We established a hypergraph learning strategy, HyperGraphComplex, for predicting protein complexes. This study effectively incorporates protein sequence features and PPIs using hypergraph learning to enhance the performance of predicting protein complexes. The performance of HyperGraphComplex outperforms multiple advanced topology-based strategies (Table 2). Bioinformatics analysis and literature validation demonstrate the effectiveness of our strategy. This study provides a new perspective on incorporating sequence features and PPI network for protein complex prediction.

We performed various ablation studies and verified the indispensable role of sequence features in model performance (Figure 4). Models that integrate protein sequence features demonstrate improved performance, benefiting from the support from sequence information. Compared to traditional graph convolution methods, hypergraph learning excels in integrating both PPI network topology and sequence information, as evidenced by comparisons with VGAE (Table 3). Additionally, we demonstrated the model’s robustness against false positive and false negative PPIs by artificially introducing or removing such noise instances from the training data (Supplementary Table 4 and 5).

We performed biological property analysis through the predicted complex dataset and observed that these complexes are not random associations but exhibit functional coherence and expression consistency, consistent with real complexes, emphasizing the reliability of our predicted complexes (Figure 5). To further elucidate the value of HyperGraphComplex, we analyzed five prediction complexes with certain biological significance. Furthermore, we confirmed the high-confidence structures of three protein complexes (CK2-Gag1 complex, SAM^Mco6^ complex, Thp3-Csn12-Sem1 complex) using AFM (Figure 7). These are promising for deepening the comprehensive understanding of cellular regulatory networks.

Current complex prediction methods derive complexes from known PPIs, making it challenging to identify all binding partners of a given protein/complex. The protein feature generation module and complex classification module of HyperGraphComplex work separately, allowing HyperGraphComplex to represent nodes in PPI networks that are not directly connected and predict the likelihood of these nodes forming a complex. Therefore, it is feasible to use the HyperGraphComplex method to identify all binding partners of a given protein/complex. For instance, our method identified 16 potential binding partners of the Cka2 subunit of the CK2 kinase complex, two of which (Gag1, score:0.94; Asf1, score:0.98) have been confirmed by independent studies [28, 66].

Nevertheless, there still exist certain limitations in the current version of HyperGraphComplex. For instance, while the HGVAE introduces hypergraph topology to obtain better protein sequence embeddings, it also obfuscates the interpretability of key sequence determinants, making it challenging to pinpoint critical interacting residues directly.

To enhance the application of our strategy, we have made all the predicted proteome-wide protein complex dataset and the corresponding program codes available on GitHub (https://github.com/LiDlab/HyperGraphComplex). We believe the community will benefit from both the hypergraph learning framework and the high-confidence proteome-wide protein complex dataset, and we anticipate that our work will attract wide attention.

**Key Points**

- This study first integrates protein sequence features and high-order PPI network topology via hypergraph learning, developing a novel computational approach HyperGraphComplex for predicting protein complexes.
- The Hypergraph Variational Autoencoder (HGVAE), an innovative model that integrates hypergraphs and variational autoencoder concepts, exhibits superior performance compared to the conventional Variational Graph Autoencoder (VGAE). This is primarily attributed to its enhanced capability to decipher intricate higher-order protein interaction patterns.
- By identifying protein complexes, HyperGraphComplex provides candidate complexes for downstream multiprotein complex structure prediction models like AFM and AF2Complex, substantially accelerating protein complex structure research and promoting understanding of biological systems.

## Supplementary Material

Supplementary_Table1-5_bbae274

## Data Availability

All the codes and datasets are packaged as π-HyperGraphComplex and available at https://github.com/LiDlab/HyperGraphComplex.

## Notes

The authors wish it to be known that, in their opinions, the first 3 authors should be regarded as joint first authors.

## Author Biographies

**Simin Xia** is a master’s student jointly trained by Anhui Medical University and National Center for Protein Science (Beijing).

**Dianke Li** is a PhD student at jointly trained by China Agricultural University and National Center for Protein Science (Beijing).

**Xinru Deng** is a master’s student at State Key Laboratory of Medical Proteomics, National Center for Protein Sciences (Beijing).

**Zhongyang Liu** is an associated professor at State Key Laboratory of Medical Proteomics, National Center for Protein Sciences (Beijing).

**Huaqing Zhu** is a professor at Anhui Medical University.

**Yuan Liu** is an assistant professor at State Key Laboratory of Medical Proteomics, National Center for Protein Sciences (Beijing).

**Dong Li** is a professor at State Key Laboratory of Medical Proteomics, National Center for Protein Sciences (Beijing).
