# Supplementary material for: Integration of protein sequence and protein–protein interaction data by hypergraph learning to identify novel protein complexes
Source: Brief Bioinform. 2024 Jun 8;25(4):bbae274. doi: 10.1093/bib/bbae274 (PMC11162299; doi:10.1093/bib/bbae274)
Supplement: Supplementary_Table1-5_bbae274 [file supplementary_table1-5_bbae274.docx]

**Supplementary Table 1.** Relationship between amino acids and CT classes.

| **CT Class** | **Amino Acid** |
| --- | --- |
| C1 | Ala, Gly, Val |
| C2 | Ile, Leu, Phe, Pro |
| C3 | Tyr, Met, Thr, Ser |
| C4 | His, Asn, Gln, Trp |
| C5 | Arg, Lys |
| C6 | Asp, Glu |
| C7 | Cys |

**Supplementary Table 2.** Performance of HyperGraphComplex in independent test dataset with 3D structures

| **PPI Dataset** | **F1-score** | **Precision** | **Recall** | **Acc** |
| --- | --- | --- | --- | --- |
| Mann | 0.842 (±0.005) | 0.833 (±0.011) | 0.853 (±0.006) | 0.731 (±0.002) |
| DIP | 0.722 (±0.008) | 0.691 (±0.013) | 0.764 (±0.012) | 0.742 (±0.005) |
| BioGRID | 0.801 (±0.006) | 0.789 (±0.009) | 0.817 (±0.005) | 0.729 (±0.002) |

| **Method** | **STRING** | | | |  | **PIPS** | | | |
| --- | --- | --- | --- | --- | --- | --- | --- | --- | --- |
|  | **F1-score** | **Precision** | **Recall** | **Acc** |  | **F1-score** | **Precision** | **Recall** | **Acc** |
| PC2P | 0.532 | **0.723** | 0.421 | 0.326 |  | 0.465 | 0.689 | 0.351 | 0.264 |
| **HyperGraphComplex** | **0.691** | 0.618 | **0.783** | **0.332** |  | **0.675** | **0.719** | **0.636** | **0.370** |

**Supplementary Table 3.** Performance on protein complex identification in two Human PPI datasets.

Note: PPI datasets and human protein complex dataset are derived from PC2P.

**Supplementary Table 4.** Robustness evaluation of HyperGraphComplex by adding False Positive PPIs

| **False Positive PPIs** | **F1-score** | **Precision** | **Recall** | **Acc** |
| --- | --- | --- | --- | --- |
| FP_0% | 0.815 (±0.002) | 0.804 (±0.006) | 0.827 (±0.004) | 0.427 (±0.002) |
| FP_10% | 0.777 (±0.003) | 0.743 (±0.007) | 0.816 (±0.004) | 0.426 (±0.002) |
| FP_20% | 0.747 (±0.003) | 0.692 (±0.008) | 0.814 (±0.005) | 0.427 (±0.003) |
| FP_30% | 0.744 (±0.004) | 0.696 (±0.009) | 0.800 (±0.006) | 0.421 (±0.003) |
| FP_40% | 0.721 (±0.002) | 0.664 (±0.005) | 0.791 (±0.005) | 0.419 (±0.002) |
| FP_50% | 0.711 (±0.003) | 0.655 (±0.006) | 0.781 (±0.004) | 0.409 (±0.002) |

**Supplementary Table 5.** Robustness evaluation of HyperGraphComplex by removing False Negative PPIs

| **False Negative PPIs** | **F1-score** | **Precision** | **Recall** | **Acc** |
| --- | --- | --- | --- | --- |
| FN_0% | 0.815 (±0.002) | 0.804 (±0.006) | 0.827 (±0.004) | 0.427 (±0.002) |
| FN_10% | 0.814 (±0.002) | 0.808 (±0.007) | 0.823 (±0.003) | 0.427 (±0.002) |
| FN_20% | 0.810 (±0.003) | 0.797 (±0.005) | 0.824 (±0.003) | 0.430 (±0.002) |
| FN_30% | 0.797 (±0.002) | 0.796 (±0.006) | 0.799 (±0.003) | 0.427 (±0.001) |
| FN_40% | 0.793 (±0.002) | 0.786 (±0.005) | 0.802 (±0.004) | 0.424 (±0.002) |
| FN_50% | 0.774 (±0.002) | 0.755 (±0.006) | 0.795 (±0.004) | 0.425 (±0.002) |
